# In Vitro Regeneration Strategies in Woody Citrus of Northeast India (*Citrus jambhiri* and *Citrus aurantifolia*)

**DOI:** 10.3390/plants15111677

**Published:** 2026-05-29

**Authors:** Priyanka Sharma, Bidhan Roy, Gopal Shukla, Monish Roy, Gadge Sushant Sundarrao, Vijay Dunna

**Affiliations:** 1 Department of Seed Science and Technology, Faculty of Agriculture, Uttar Banga Krishi Viswavidyalaya, Pundibari, Cooch Behar 736165, India; bcroy10@yahoo.com (B.R.); roymonish17@gmail.com (M.R.); sushantgadge4@gmail.com (G.S.S.); 2 AICRP on Seed (Crops), Department of Plant Breeding and Genetics, Assam Agricultural University, Jorhat 785013, India; 3 Department of Forestry, North-Eastern Hill University, Tura Campus, Tura 794002, India; gopal@nehu.ac.in; 4 Division of Seed Science and Technology, ICAR-Indian Agricultural Research Institute, Pusa Campus, New Delhi 110012, India; dunna.vijay@icar.org.in

**Keywords:** *Citrus jambhiri*, *Citrus aurantifolia*, callus induction, regeneration, organogenesis, rhizogenesis

## Abstract

Since the status of *C. jambhiri* is “rare” and *C. aurantifolia* is endowed with folk medicinal properties, our study aimed at producing true-to-type seedlings for further conservation by using PGRs, ferrous sulfate, and casein hydrolysate in MS medium to induce in vitro germination and “callus” formation from seeds of *C. jambhiri* Lush. It also focused on evaluation of suitable species and the best type of explant for organogenesis. The present study was undertaken to develop an efficient micropropagation protocol for *C. jambhiri* Lush. and *C. aurantifolia*. The frequency of callus induction increased to its maximum when 2.0 mg/L 2,4-D and 1.0 mg/L picloram were added individually. The calli derived from 2,4-D exhibited maximum regeneration potentiality. In addition, sucrose (30 g/L), dextrose (60 g/L), and coconut water (10 mL/L and 15 mL/L) also enhanced callus induction. Regarding heavy metals, 100 ppm of Fe_2_SO_4_ exhibited maximum germination percentage (84.33%) from seeds of *C. jambhiri* Lush. However, maximum callus induction (50.00%) was induced from the seeds of *C. aurantifolia* incubated in Fe_2_SO_4_ (400 ppm). The maximum number of shoots per callus was produced (5.13) with the addition of 200 mg/L casein hydrolysate in the callus-induction medium. Pearson correlation analysis revealed a positive and significant association of the number of plantlets/calluses with shoot length and regeneration percentage, respectively. It was phenotypically observed that the tissue culture traits performed better from explants derived from *C. jambhiri* Lush. than *C. aurantifolia* when subjected to varying concentrations of PGRs, carbon sources, organic adjuvants, and heavy metals, respectively.

## 1. Introduction

The northeastern region of India, which is the center of origin, is endowed with a wide diversity of citrus, including wild and endangered species. However, due to overexploitation and extensive degradation of its natural habitat, it has resulted in a decline in the genetic diversity of citrus [1]. Reports from the India Biodiversity Portal (2016) [2] signify that rough lemon (*C. jambhiri*), although it exists in its natural habitat, is currently categorized as “rare” in the region. *Citrus aurantifolia* (acid lime), due to its folk medicinal properties viz., antibacterial, anticancer, antidiabetic, antifungal, antihypertensive, anti-inflammatory, and antioxidant effects, has been extensively utilized. Therefore, with respect to the requirement for pharmacological purposes and the restoration of degraded-range lands, it has become increasingly important to develop techniques for the large-scale multiplication of certain genotypes of citrus species through PTC [3].

Plant regeneration using somatic tissues is considered to be a successful method for propagating clonal plants. However, developing protocols for successful plant tissue culture is complicated due to genotype variations and seasonal dependency, as the explant’s regeneration capability is associated with the donor plant’s physiological conditions and varies from year to year, along with other interacting factors. In every plant cell culture condition, the quality of the final product is determined by the plant raw material, culture conditions, and composition of the culture media [4]. Based on the medium’s nutritional balance, plants control their growth pathway and, more specifically, high levels of chloride (Cl) and nitrogen (N) inhibit rapid cell division in vitro [5]. Hyperhydricity is a major problem regarding endogenous hormonal balance in plant micropropagation [6]. The selection of organic adjuvants, growth regulators, carbon sources, heavy metals, and nutritional components is one of the most important factors influencing the development and morphogenesis of plant tissues in culture [7]. In addition, the main factor affecting the rooting of in vitro seedlings depends on the effect of exogenous PGRs and MS medium is not suitable for rooting [8]. The success of callus induction and subsequent plantlet regeneration is increased when nutritional requirements are available in optimum concentrations for the best in vitro tissue growth [9].

Plant growth regulators (PGRs) are a diverse group of organic compounds that regulate the morphological, biochemical, and physiological aspects of plant development and can either promote or inhibit plant growth [10]. Plant growth and development can be effectively enhanced by a range of organic growth supplements, including pineapple pulp, banana pulp, tomato juice, papaya juice, coconut water, potato homogenate, and yeast extract [11]. The types and concentrations of organic nutrients required for the success of in vitro cultures vary depending on the species and genotype [12]. In tissue culture media, cytokinins are the most often added growth regulators. “Cytokinin” can be found in both natural and synthetic sources, including tender coconut water and kinetin; however, kinetin is significantly effective at lower concentrations and tender coconut water at higher concentrations [13]. Sucrose has been widely used in the majority of studies on in vitro shoot induction and development, root induction, callogenesis, embryogenesis, and regeneration [14]. Monosaccharide hexoses (glucose, fructose, galactose, and mannose), pentoses (arabinose, ribose, and xylose), disaccharides (maltose, lactose, cellobiose, and trehalose), and tri-saccharides (raffinose) are some additional major sugars that plants can use; however, glucose and fructose are rapidly degraded [15].

Metals are typically needed in very low concentrations for plant growth and development, but can become phytotoxic when present in excessive concentrations [16]. Although Cu and Zn are necessary for plant growth and development, they can be harmful at higher concentrations [17,18]. Fe, Al, and Ni were examined in our study for callus induction, respectively. When iron (Fe) at a very low concentration was used for the study, we could observe photosynthetically green callus at the beginning that helped in chlorophyll biosynthesis. Similarly, Al can act as a stress signal, enhancing phenolic production and stimulating callus induction via stress-mediated differentiation. Nickel at optimum concentrations supports callus growth by enabling proper nitrogen utilization in the form of urea. Heavy metals at higher concentrations disrupt many plants’ metabolic processes, which hinders the plants’ growth and development. The selection of plants resistant to specific metal ions is made possible via in vitro plant tissue culture. However, no previous reports have been found to date using ferrous sulfate, nickel chloride, and aluminum sulfate in citrus tissue culture. In the present study, we examined and emphasized the enhancement of callogenesis and “rhizogenesis” using different concentrations and combinations of picloram, casein hydrolysate, ferrous sulfate, aluminum sulfate, and nickel chloride in nutrient medium, and to the best of our knowledge, scattered information is available to date. Casein hydrolases are used to mitigate the adverse effects of abiotic plant stress caused by heavy metals, drought, and salinity [19]. Nutrient medium was used in our study since a specific nutrient balance requirement is essential for soft callus induction with rapid post-mitotic cell expansion [20]. Due to the flexibility of plant development by either callus induction or plantlet regeneration, this formulation, called MS, had prior support and demonstrated promising results in preliminary research within specific plant groupings. Because of its high rates of hyperhydricity, poor adaptability of the regenerated plants, and decreased shoot multiplication, the media is not appropriate for rooting for commercial micropropagation [21]. In this study, optimization of an in vitro regeneration protocol using mature seeds, stem sections, and leaves was performed. This study also highlights the identification of suitable citrus species and selection of the best type of explants derived from in vitro grown seedlings.

## 2. Results

### 2.1. Exogenous PGRs for Germination and Callus Induction from Leaf and Stem Segments

**Figure 1:**
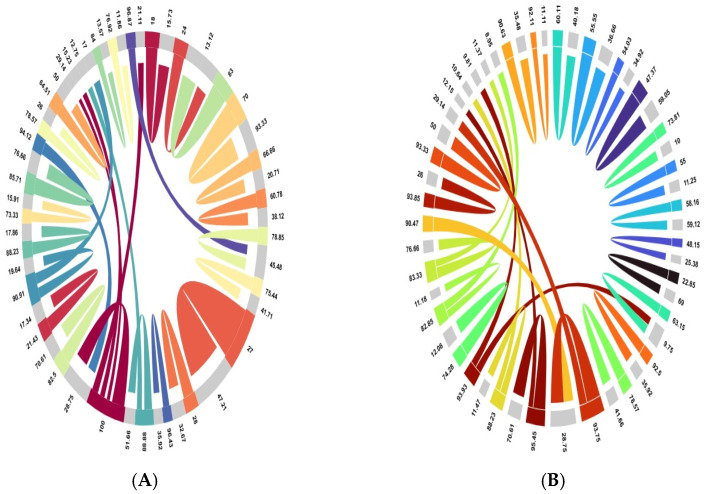
(**A**,**B**) Chord diagram indicating the relationship of visual representation of mean values in three replications of callus-induction percentage with regeneration percentage and number of plantlets/calluses in (**A**) *C. jambhiri* and (**B**) *C. aurantifolia*. Relationships are drawn as arcs whose width represents their values.

**Figure 2:**
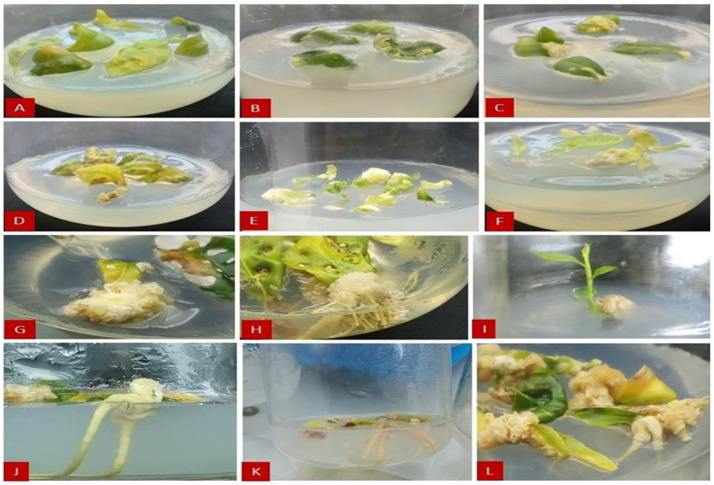
Callus induction and regeneration of *C. jambhiri* from leaf. (**A**) Leaves inoculated on callus-induction medium. (**B**) Calli induced on MS + 2,4-D 2.0 mg/L. (**C**) Calli induced on MS + 2,4-D 1.0 mg/L + NAA 0.50 mg/L. (**D**) Calli induced on MS + TDZ 0.25 mg/L. (**E**) Calli induced on MS + 2,4-D 1.0 mg/L + NAA 0.50 mg/L. (**F**) Calli induced on MS + 2,4-D 2.0 mg/L + NAA 0.50 mg/L. (**G**) Embryogenic leaf callus. (**H**) Embryogenic leaf callus showing rhizogenesis. (**I**) Regenerated plantlet from the leaf callus. (**J**,**K**) Rhizogenesis from leaf calli. (**L**) Rhizogenesis from leaf calli.

**Figure 3:**
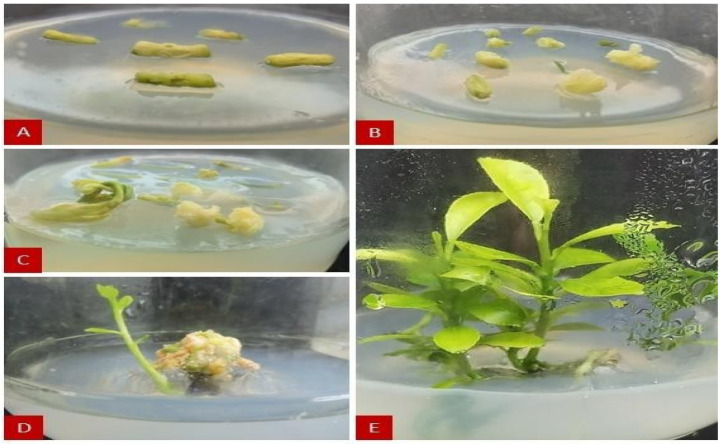
Callus induction and regeneration from the stem sections derived from the callus of *C. aurantifolia*. (**A**) Stem sections inoculated on callus-induction medium. (**B**) Callus induction from stem sections. (**C**) Callus proliferation from stem sections. (**D**) Plantlet regeneration from callus. (**E**) in vitro-established plantlets.

**Figure 4:**
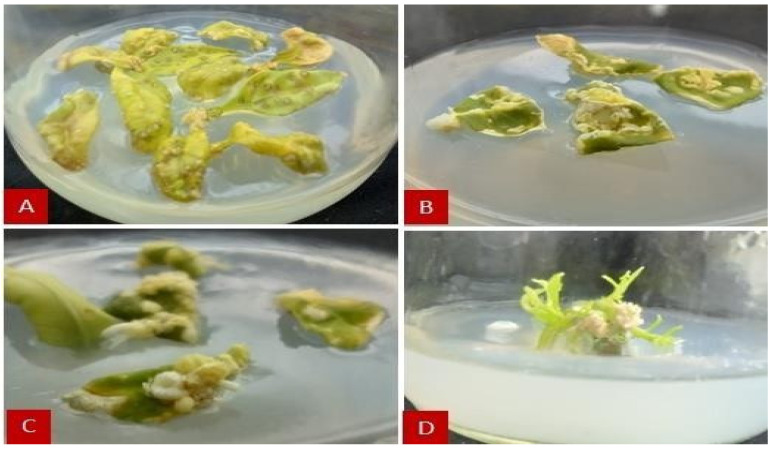
Callus induction and regeneration from leaf-derived callus of *C. aurantifolia*. (**A**) Leaves inoculated on callus-induction medium. (**B**) Callus initiation on leaves. (**C**) Proliferation of calli on callus-induction medium. (**D**) Regeneration of plantlets from leaf callus.

Analysis of variance indicated highly significant variations among the treatments and the explants with respect to seed germination, callus induction, plantlet regeneration, number of shoots per responsive callus, number of roots per shoot, and shoot length in *C. jambhiri*, respectively (Table S1). Germination percentage was evaluated only from the mature seeds, as its values are represented in Figure 1A. When Dicamba (1.0 mg/L) was added, the maximum germination percentage (95.00%) was recorded; however, adding 0.25 mg/L TDZ exhibited a minimum value of 11.00%. The cultured leaf segments produced embryogenic callus and showed “rhizogenesis” in all areas when subjected to 2.0 mg/L 2,4-D, while 1.0 mg/L 2,4-D along with 0.50 mg/L NAA exhibited maximum callus from “stem” segments, and 2.0 mg/L 2,4-D combined with 0.50 mg/L NAA, respectively (Figure 2). Similarly, in *C. aurantifolia*, the performance of callus induced from leaf and stem segments could also be observed as indicated in Figure 3 and Figure 4. However, leaf and stem segments derived from in vitro-grown seedlings were incubated for callus induction and plantlet regeneration. Leaf segments exhibited 100% callus in a combined treatment of 1.0 mg/L 2,4-D along with 0.50 mg/L NAA, as well as 2.0 mg/L 2,4-D combined with 0.50 mg/L NAA and 0.50 mg/L TDZ (Figure 1B and Figure 3). On the contrary, stem segments exhibited comparatively less callus induction than leaf segments treated with 1.0 mg/L along with 0.50 mg/L TDZ (Figure 4). The highest mean value (74.46%) could be observed from 1.0 mg/L 2,4-D subjected to exogenous PGRs (Table S2).

### 2.2. PGRs for Plantlet Regeneration Efficiency

**Figure 5:**
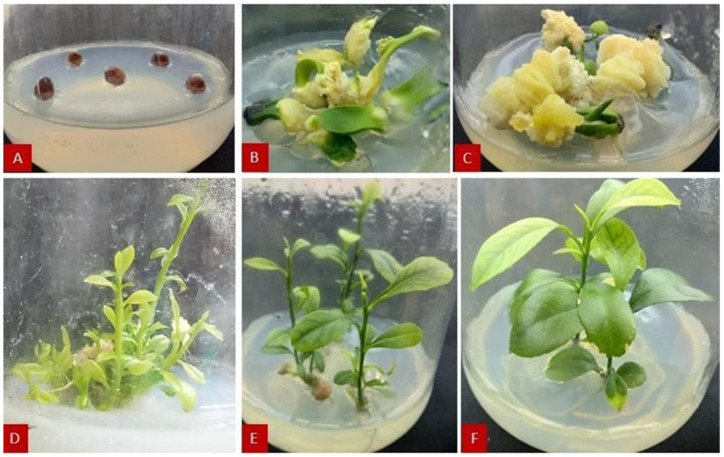
Callus induction and regeneration from mature seed-derived callus of *C. aurantifolia*. (**A**) Inoculated seeds on callus induction medium; (**B**) Callus induction at the base of cotyledons of germinating seeds; (**C**) Well grown callus on callus induction medium; (**D**) Multiple shoots regeneration from callus; (**E**) Individual regenerated shoots inoculated on rooting medium for further growth of shoots and simultaneous root regeneration; (**F**) Well grown shoots with roots.

**Figure 6:**
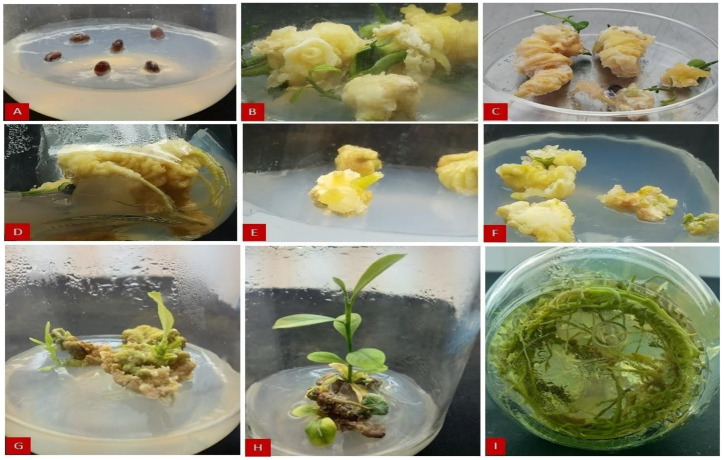
Callus induction as influenced by different concentrations of Dicamba and subsequent regeneration of *C. jambhiri* from mature seeds. (**A**) Matured seeds inoculated on callus-induction medium. (**B**,**C**) Calli induced on MS + 1.0 mg/L Dicamba. (**D**) Calli induced on MS + 1.0 mg/L Dicamba. (**E**) Embryogenic calli. (**F**) Embryogenic calli. (**G**) Regenerated plantlets on MS + 1.0 mg/L kinetin + 1.00 mg/L BAP + 0.5 mg/L NAA. (**H**) Regenerated plantlets on MS + 1.0 mg/of kinetin + 1.00 mg/L BAP + 0.5 mg/L NAA. (**I**) Rooted regenerated plantlets.

The embryogenic calli subcultured in 2.0 mg/L 2,4-D and 0.50 mg/L NAA exhibited maximum regeneration efficiency (59.72%), as indicated in Table S3. Leaf segments produced the highest percentage of callus; however, the regeneration percentage was minimal due to poor callus health (Figure 5). Maximum regeneration (36.31%) was observed in callus obtained from the stem (Table S2). Our findings revealed that the maximum mean value for the number of shoots per callus was recorded when the calli were induced from mature seed (2.84) (Table S2). We observed that the majority of calli obtained from leaves showed deficiency symptoms of shoot regrowth. Calli derived from leaves indicated poor health. On the regeneration medium, most of them appeared discolored, and the callus was likewise smaller in size and fragile. In terms of the number of roots per plantlet, the maximum value (6.86) was obtained from the matured seed callus incubated in 2.0 mg/L 2,4-D along with 0.50 mg/L as indicated in Table S3 and Figure 6. In *C. aurantifolia*, explants with the highest mean number of roots per plantlet (3.92 roots/plantlet) were derived from the calli induced from mature seeds (Table S4). The highest shoot length (4.32 cm) was obtained from the seed calli incubated in 1.0 mg/L 2,4-D. The mean value indicated that the maximum value of shoot length (4.32 cm) could be observed from the regenerated plantlets incubated in 1.0 mg/L 2,4-D as indicated in Table S5. The shoot length of every regenerated plantlet was higher than 2.00 cm, and the maximum shoot length of 3.67 cm was obtained from the regenerated plantlets of matured seeds (Table S4).

### 2.3. Organic Adjuvants for Germination, Callus Induction, and Plantlet Regeneration Efficiency

**Figure 7:**
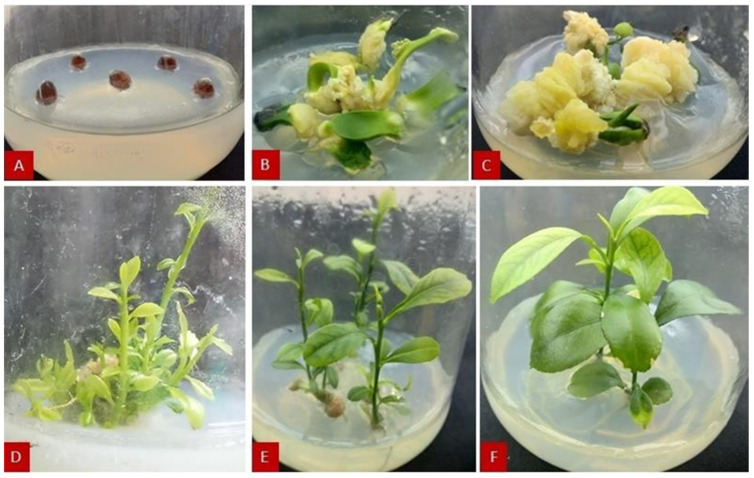
Callus induction and regeneration from mature seed-derived callus of *C. aurantifolia*. (**A**) Inoculated seeds on callus-induction medium. (**B**) Callus induction at the base of cotyledons of germinating seeds. (**C**) Well-grown callus on callus-induction medium. (**D**) Multiple shoots regeneration from callus. (**E**) Individual regenerated shoots inoculated on rooting medium for further growth of shoots and simultaneous root regeneration. (**F**) Well-grown shoots with roots.

Significant variations with respect to in vitro morphogenetic responses of *C. jambhiri* and *C. aurantifolia* are indicated in Table S6. However, no statistically significant differences across citrus species in terms of the number of shoots per callus were observed (Tables S7 and S8). The results reflected a better performance in germination percentage with a higher concentration of casein hydrolysate (200 mg/L) in *C. jambhiri* (Table 1). Medium fortified with yeast extract (600 mg/L) was found to be the best for in vitro germination of seeds (Table S7). Among the species, the mean value of maximum germination percentage of 63.48% was recorded in *C. aurantifolia* (Table S9). When 100 mg/L casein hydrolysate was added, matured seeds of *C. jambhiri* exhibited a maximum callus-induction ability of 60.00% (Figure 7). Thus, *C. jambhiri* was shown to give higher results (31.57%) than *C. aurantifolia* (20.40%) among the species (Table S9). The highest plant regeneration efficiency and number of plantlets/calluses were obtained from the callus derived from matured seeds incubated in 50 mg/L casein hydrolysate (Table 1). With 200 mg/L casein hydrolysate added to the callus-induction medium, the maximum number of shoots per callus was produced (5.13) in *C. jambhiri*. Matured seeds incubated in 100 mg/L casein hydrolysate exhibited the maximum germination percentage (98.00%) in *C. aurantifolia*. The maximum number of roots per shoot (8.76) could be observed from the callus induced in 100 mg/L casein hydrolysate. The similar treatment exhibited the highest number of roots (5.55). (Table S8). Comparative analysis of effects showed that *C. aurantifolia* indicated the maximum efficiency of root regeneration per shoot (2.97) compared to *C. jambhiri* (2.18), respectively (Table S9). The longest shoot (5.29 cm) was recorded when the callus-induction medium was supplemented with 50 mg/L casein hydrolysate (Table S8).

### 2.4. Carbon Sources for Callus Induction and Plantlet Regeneration

Highly significant variations were observed among treatments, species, and their interaction with tissue culture traits; however, the number of roots per shoot and the length of did not significantly differ among species or when species and treatments were combined (Table S10). In total, 60 g/L sucrose-supplemented callus-induction medium exhibited the highest germination percentage of 98.00% in *C. jambhiri* (Table S11). Sucrose at a concentration of 60 g/L and 30 g/L (67.17%) was found to be the best treatment for in vitro germination of mature seeds. *C. jambhiri* exhibited with the highest callus-induction efficiency (Table S12), and maximum regeneration was observed in calli of *C. jambhiri* when regeneration medium was added with 30 g/L sucrose. Our findings revealed that *C. jambhiri* exhibited a stronger response to the effects of carbon sources (41.87%) as shown in Table S13. Regarding the number of shoots per callus, 60 g/L sucrose exhibited the highest value of 9.33 in *C. jambhiri* (Table S11). The average data revealed that 60 g/L sucrose showed maximum effectiveness in enhancing the number of shoots per callus (4.21 shoots per callus) (Table S13). *C. jambhiri* responded better regarding the number of shoots per callus as indicated in Table S13. With respect to the number of roots per shoot, 60 g/L sucrose showed the maximum number of roots per shoot (3.28) in *C. jambhiri* (Table 2). The highest shoot length (3.76 cm) was observed from the regenerated plantlet incubated in a 60 g/L sucrose-supplemented callus-induction medium (Table S12). Regarding the species effect, *C. jambhiri* exhibited the highest shoot length (Table S13).

### 2.5. Heavy Metals for Callus Induction and Plantlet Regeneration

When 100 ppm of Fe_2_SO_4_ was added to the MS medium, an average intensity of germination (84.33%) was obtained in *C. jambhiri* (Tables 3 and S15). It was interesting to observe that the 100 ppm of Fe_2_(SO_4_)_3_ exhibited better germination rates (68.78%) than the control, as indicated in Table S15. Maximum callus induction was observed when the medium was fortified with 400 ppm of Fe_2_(SO_4_)_3_ (50.00%), as indicated in Table S15. *C. aurantifolia* exhibited the highest efficiency of callus induction (22.03%) as shown in Table S16. Fe_2_(SO_4_)_3_-supplemented MS medium produced the highest plantlet regeneration response (39.23%) as indicated in Table S15. Our findings revealed that *C. aurantifolia* responded much better than *C. jambhiri* regarding callus induction (26.25%) and plantlet regeneration (17.64%), respectively as indicated in Table S16.

### 2.6. Interpretation of Pearson Correlation Analysis PGRs

**Figure 8:**
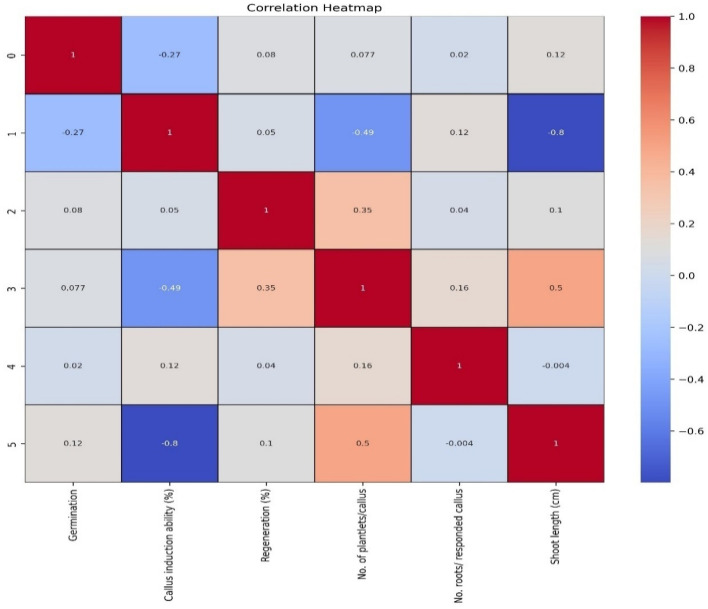
A correlation heatmap was produced, indicating the non-significant association with callus-induction ability, regeneration percentage, shoot length, number of plantlets/callus, and number of roots responded/callus in *C. jambhiri*.

**Figure 9:**
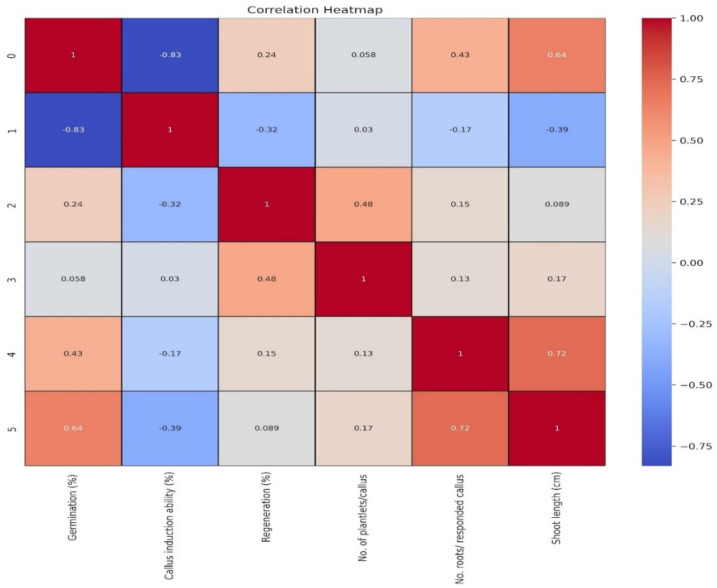
A correlation heatmap was produced, indicating the non-significant association with callus-induction ability, regeneration percentage, number of plantlets per callus, number of roots responded/callus, and shoot length in *C. aurantifolia*.

In *C. jambhiri*, callus-induction ability exhibited a positive and non-significant correlation with the number of roots responding/callus (0.122 NS) and regeneration percentage (0.05 NS). The number of plantlets/calluses had a positive and significant relationship with shoot length (cm) (0.5 **). However, the number of roots responding/callus was positive and non-significant with the number of plantlets/callus (0.16 NS), callus-induction ability percentage (0.122 NS), regeneration percentage (0.04 NS), and germination (0.02 NS). In *C. aurantifolia*, with respect to germination percentage, it had a positive and significant relationship with shoot length (cm) (0.643 **) and number of roots responding/callus (0.43 *) as indicated in Figure 8. Callus-induction ability showed a positive and non-significant association with respect to the number of plantlets callus^−1^ (0.03 NS). The number of plantlets/calluses exhibited a positive and significant relationship with regeneration percentage (0.476 **). The number of roots responding/callus showed a significant association with shoot length (cm) (0.724 **) and germination percentage (0.43 *), respectively, as indicated in Figure 9.

## 3. Discussion

### 3.1. Influence of Exogenous PGRs on Plantlet Regeneration Efficiency

The significance of the citrus sector and the introduction of enhanced genotypes highlight the need for modern techniques to quickly propagate novel and promising plant material [22]. Rootstock seeds are sometimes unavailable, and some potentially intriguing rootstocks do not generate enough nucellar seedlings due to low levels of polyembryony [23]. Somatic hybrid rootstocks can be propagated by micropropagation, particularly those with trifoliate orange parentage [24]. Micropropagation methods could rapidly provide budwood in cases when existing orchards are being grafted onto new cultivars that are scarce. Citrus rootstock propagation in vitro techniques that are quick and affordable would be highly desirable in this context. In addition, more consistent disease-free planting material may be produced since the inability to supply seeds would not be a limiting factor in plant production [25]. Studies on the regeneration efficiency of citrus species revealed the significant influence of cytokinin type and concentration, as well as cytokinin–auxin ratios, on regeneration from various explant types [26]. Apart from the selection of genotypes and explants, other factors such as PGRs, organic adjuvants, carbon sources, and heavy metals influence the germination potential, callus-induction efficiency, and regeneration response of endangered citrus species. Exogenous hormone addition and tissue culture response to these hormones are necessary for plant regeneration in vitro [27]. PGRs, particularly auxins, enhance root formation, while cytokinins help in cell division [28]. For *Citrus latifolia*, the optimal medium for regeneration from nodal explants and elongation was supplemented with 1 mg/L BA and 0.01 mg/L NAA [29]. 2,4-D is useful in the development of somatic embryos, and its presence in culture media appears to promote somatic embryogenesis in sweet orange [30]. Citrus epicotyls have predominantly been used to standardize regeneration protocols [31], and matured shoots containing 1.0 cm internodes have also been utilized for the regeneration of mature tissues in citrus in our findings. This is because their parenchymatous cells are highly responsive, genetically stable, easy to culture, and capable of direct shoot organogenesis with reduced genetic instability. Unlike leaf or root explants, internodal tissues often show a higher frequency of “adventitious shoot bud initiation” in citrus. In our findings, callus obtained from the treatments supplemented with 2.0 mg/L 2,4-D, along with 0.50 mg/L NAA and 0.25 mg/L TDZ, was observed to be in good health with rapid proliferation of root and shoot that resulted in high regeneration efficiency. Hypothetically, lower concentrations of auxins, probably 2,4-D, NAA, and TDZ, allow for balanced and regulated hormonal action that reduces the risk associated with excessive callus formation or abnormal tissue growth. Auxins control the direction of morphogenesis and encourage the development of calli, cell suspensions, and organs, primarily in conjunction with cytokinins [32]. They contribute to the development of meristems that give rise to either defined organs or unorganized tissue because they can start cell division [32]. This leads to more efficient and rapid root and shoot proliferation. The choice of auxins and their concentrations depend upon the type of growth or development required. Auxin is essential for the development of meristems [33] and initiates cell division and leaf commencement before lateral root initiation [34]. Auxin at an optimal concentration can help in initiating plant roots; exogenous auxin can promote GA production and auxin-triggered pathways while suppressing ABA and ET biosynthesis [35]. In addition, it can act synergistically to promote early cell division and the formation of primordia. In plants, endogenous auxin is responsible for both shoot and root formation. Depending on the explant source, stem segments derived from aseptic seedlings supplemented with 0.5 mg/L NAA, along with 1.0 mg/L BAP, produced 4.00 ± 0.69 shoots per explant in *Citrus mitis* [36]. Our results are in contrast with those of [37,38] who observed that callus derived from cotyledonary explants of *C. jambhiri* and stem segments of *C. sinensis* induced on 2,4-D (2.0 mg/L and 4.53 mg/L) and NAA (5.37 mg/L)-supplemented medium exhibited robust regeneration potential and number of shoots/callus (6). Hypothetically, an efficient regeneration protocol would substantially enhance the rapid proliferation of roots and shoots and establish a basis for a citrus genetic transformation system. It is commonly known that the dormant meristematic zone on the axillary bud area of the node initiates shoot organogenesis or differentiation when the cytokinin-to-auxin ratio is appropriate [39]. Effective regeneration is contingent upon a suitable micropropagation protocol, encompassing explant kinds, medium compositions, and culture conditions [40]. Plant regeneration in tissue culture mostly occurs by de novo organogenesis and somatic embryogenesis [41]. Auxin activity is inherently defined by its source, transport, and local accumulation. The root is the site of formation of an endogenous auxin gradient. Therefore, exogenous auxin cannot directly affect rooting since it may disturb a gradient. Different levels of auxins have a distinct efficiency for root induction, which coincides with the variable affinities of auxin receptors implicated in rhizome development and varying concentrations of free auxin reaching target cells. Different auxin concentrations, including α-naphthalene acetic acid and indole-3-acetic acid (IAA), are also crucial for encouraging adventitious root development in tissue culture [42]. Picloram is frequently utilized for plantlet regeneration in several palm species [43]. Picloram is effective in inducing callus formation from explants, since the addition of 1.0 mg/L picloram subsequently enhanced callus-induction ability up to 90.91% from the stem and 60.78% from the matured seeds of *C. jambhiri*, respectively, in our findings. When used at an optimum concentration, picloram enhances the regeneration efficiency of explants, which leads to the production of multiple shoots or embryos from a single explant. The first report on the addition of picloram to MS medium for enhanced induction of organogenic calli and its high frequency indirect re generation from leaves was observed in *Gerbera jamesonii* [44]. Picloram (1.0 mg/L) substantially enhanced callus-induction efficiency up to 43.7% in other crops [45]. However, as per the literature search in several databases, no reports have been found to date. Therefore, to the best of our knowledge, scattered information on the use of picloram for enhanced callus-induction ability and high-frequency plantlet regeneration is available. An optimal concentration of 2,4-D is commonly employed to induce calli from explants of in vitro-derived seedlings under controlled conditions [46]. Leaf explants derived from in vitro grown seedlings of *C. sinensis* exhibited a maximum callus percentage of 98.33% when incubated in 4.53 mg/L 2,4-D along with 5.37 mg/L NAA, respectively [47]. Varying species or tissues have varying optimal 2,4-D concentrations, which have an impact on callus development. A low concentration encourages the production of embryonic callus, while a high concentration prevents this process [48]. Well-developed somatic embryos of *C. jambhiri* incubated in 1.0 mg/L 2,4-D along with 1.0 mg/L BAP rapidly produced a higher percentage of callus [49]. The medium containing 2.0 mg/L 2,4-D alone also recorded the maximum efficiency of callus induction (91.66%), respectively [50]. Cotyledons and stem segments of *C. reticulata* incubated in 1.0 mg/L or 2.0 mg/L 2,4-D exhibited 100% callus efficiency [51]. In our findings, 0.25 mg/L TDZ recorded the maximum number of plantlets/calluses, while the rooting efficiency with a value of 12 was due to the effect of 2,4-D 1.0 mg/L, along with 0.50 mg/L NAA in *C. jambhiri*. TDZ is effective in the initiation of the regenerative response to calli. The calli of *Citrus indica* exhibited the highest mean shoot induction frequency, 4.9 shoots/callus, when supplemented with 0.5 mg/L BAP along with 0.25 mg/L TDZ and 0.25 mg/L NAA, respectively, which is in accordance with our findings [52]. As in the present study, other workers have reported the use of combinations of cytokinins and auxins for inducing shoot regeneration where the concentrations of exogenous cytokinins were higher than those of auxins [53]. A shoot length of 8.81 cm was observed due to the supplementation of 1.0 mg/L 2,4-D. In indirect regeneration, shoot length increases primarily during the elongation phase, which is promoted by lowering cytokinin levels. In *C. indica*, shoots were regenerated and elongated at a length of 4.63 cm due to the combined effect of BAP, kinetin, and NAA [52]. Similarly, regarding *C. aurantifolia*, an increase in germination percentage could be observed in seeds supplemented with 1.0 mg/L 2,4-D, while maximum calli were induced in 2,4-D 2.0 mg/L along with NAA 0.50 mg/L. TDZ at a concentration of 0.25 mg/L regenerated maximum calli. Similarly, 2,4-D 1.0 mg/L, along with NAA 0.50 mg/L, exhibited maximum plantlets/callus with robust root and shoot proliferations, respectively, supplemented with 2,4-D 1.0 mg/L. In the vast majority of cultivated explants, the majority of the shoots grow back from the callus’s middle cells or peripheral cells. According to histological analyses of callus cultures, especially in *Citrus madurensis*, the middle and outer peripheral cells are of parenchymatous and meristematic origin and aid in further participation in cell division and differentiation [54]. The occurrence of redifferentiation in the formation of shoots was obtained when the calli of *Citrus grandis*, *Citrus sinensis*, and *Citrus aurantifolia* were cultured on BAP- and NAA-supplemented MS medium [55]. Our findings corroborate with other researchers’ reports on indirect organogenesis (44%, 40%, and 48%) and were also obtained when the callus was inoculated with 3 mg/L BAP along with 0.5 mg/L NAA [56].

### 3.2. Influence of Organic Adjuvants for Germination, Callus Induction, and Plantlet Regeneration Efficiency

Organic adjuvants not only enhance tissue culture response but also boost in vitro plant growth and morphogenesis. The level of organic adjuvants required for efficient plant tissue culture is primarily dependent upon the plant species and the genotype. Our findings revealed that coconut water significantly enhanced callus induction, which corroborates the results of [57] that green calli incubated in coconut water (5 mg/L, 10 mg/L) extracted from the Pink Dwarf Variety significantly enhanced embryogenesis and plantlet regeneration in *Oryza sativa* L., var. Karnal local [58]. The concentrations of organic nutrients required for in vitro culture success vary depending on the species and genotype [59]. Adding organic growth enhancers to culture media can promote tissue growth and boost the number of roots, shoots, and leaves. Supplementing with organic growth accelerates the establishment and expansion of cultures and produces vigorous plants [59]. Our findings revealed that yeast extract, casein hydrolysate, and coconut water robustly improved callus induction and subsequent regeneration of plantlets, respectively, in *C. jambhiri* and *C. aurantifolia*. However, coconut water was observed to be more effective in enhancing tissue culture responses compared to yeast extract and casein hydrolysate. This is because the major components contained in coconut water are cytokinins, which improve organogenesis [59]. The tender fleshy portion of the coconut was used in orchid tissue culture to study its effects on orchid seed germination [60]. The addition of 400 mg/L casein hydrolysate significantly enhanced the multiplication of embryos derived from *C. aurantium* [61]. It also helped to promote embryo growth and development when added to culture medium [62]. Interestingly, in our findings, 50 mg/L casein hydrolysate was found to be more effective in regeneration efficiency. A study conducted by a few researchers opined that casein hydrolysate is more effective than amino acids with respect to shoot regeneration [63], since the addition of 150 mg/L casein hydrolysate, along with juice vesicles of *C. reticulata* to MS medium, significantly enhanced callus induction and plantlet regeneration in mandarin orange. Casein hydrolysate acts as a chelating and complexing agent, thereby keeping essential mineral ions, particularly calcium and zinc, soluble and available for plant uptake and prevents the formation of insoluble mineral precipitates. Our findings revealed that the number of plantlets and roots increased to the maximum when supplemented with 100 mg/L casein hydrolysate. However, a lower concentration of 50 mg/L enhanced the shoot length of both species. In *C. reticulata* Blanco, maximum shoot proliferation could be enhanced by the addition of 100 mg/L casein hydrolysate along with 25 mg/L glutamine and 50 mg/L adenine sulfate, which conforms with our findings [64].

### 3.3. Influence of Carbon Sources for Callus Induction and Plantlet Regeneration

One of the key factors influencing plant regeneration in culture media is the carbon source. The most often employed sugars in plant tissue culture are glucose, sucrose, and maltose. Sugar gives the culture energy and serves as the primary regulator of the permeability environment. Somatic embryo maturation can be accelerated by small sugar molecules that can enter live cells and dehydrate somatic embryos [65]. Thus, excessive concentrations of carbon sources might be detrimental, hindering the growth and development of in vitro-derived plantlets [66]. In our findings, we could observe maximum intensity of regeneration efficiency at 30 g/L sucrose in *C. jambhiri*. We could also observe the maximum number of plantlets/calluses and rooting at 60 g/L sucrose. Shoot length was increased due to the effect of a higher concentration of dextrose at 60 g/L. Somatic embryo development benefits from a low sucrose concentration during somatic embryogenesis [67]. Explants cultured on lower concentrations of sucrose, viz., 30 g/L to 40 g/L, efficiently enhanced shoot proliferations in Carrizo citrange as compared to higher concentrations, viz., 50 g/L to 60 g/L, respectively [68]. For adventitious shoot formation in *C. aurantifolia*, the optimal concentrations of sucrose were found to be 30 g/L to 50 g/L rather than 60 g/L. Some cells undergo dedifferentiation and redifferentiation, which leads to adventitious shoot development. Adventitious shoots can originate from the surface of the callus or directly from explants that possess external phloem and surface cambium. Sometimes, callus generates adventitious shoots after several subcultures [69]. A medium’s optimal sucrose concentration should be sufficient to provide the fundamental energy needs for cell division and differentiation without having an adverse osmotic effect on the development of shoots [70]. This suggests that sucrose functions as an osmoticum in addition to being a carbon energy source in a medium and that the induction and growth of shoots are influenced by a variety of sucrose concentrations [71].

### 3.4. Influence of Heavy Metals on Callus Induction and Plantlet Regeneration

The ability of explants to regenerate can also be impacted by additional elements introduced to the culture medium, such as metal ions [19]. Regarding the addition of heavy metals, viz., aluminum sulfate, ferrous sulfate, and nickel chloride, the findings of the previous study enhanced tissue culture responses relative to controls with reduced metal toxicity levels [67]. Our findings confirmed that a lower concentration of Fe_2_(SO_4_)_3_ (100 ppm) facilitated plantlet regeneration and the number of plantlets/calluses. Interestingly, rooting was observed in the control and at a higher concentration of Fe_2_(SO_4_)_3_ (400 ppm), and shoot length increased. The results of our study corroborate the findings of those developed in rice [66] ZnSO_4_ at 100 µM in the Murashige and Skoog (MS) basal medium produced vigorous shoot development (9) with a maximum shoot length of 7.2 cm. In addition, a lower concentration of CuSO_4_ (50 µM) produced the maximum shoot number (7) with healthy shoots and leaves [68].

## 4. Materials and Methods

### 4.1. Collection of Plant Material and In Vitro Seed Germination

**Figure 10:**
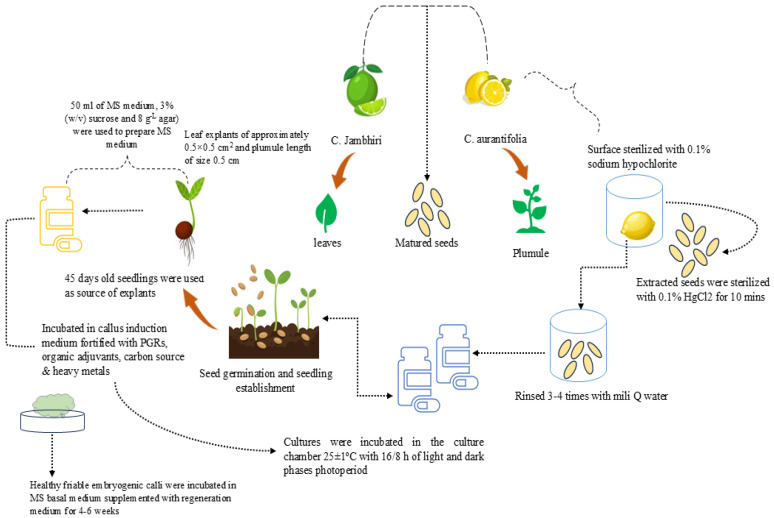
Graphical illustration of methodology indicating collection of donor plant material for in vitro seed germination, callus induction, plantlet regeneration, and hardening for complete plant establishment.

Mature fruits of *C. jambhiri* Lush. and *C. aurantifolia* was obtained from the AAU Citrus and Plantation Crops Research Station, Tinsukia, Assam, India (CPCRS). Fruits were surface-sterilized for 2 min in a sodium hypochlorite solution (0.1%) (Himedia Laboratories Pvt. Ltd., Thane, India). Surface-sterilized fruits were bisected using a sharp knife for manual removal of seeds. The extracted seeds were disinfected (under a laminar airflow chamber) with 0.1% HgCl_2_ (Himedia Laboratories Pvt. Ltd., Thane, India) for 10 min and rinsed 4–5 times with sterile Milli-Q-water (Millipore, Bangalore, India) followed by de-coating before inoculation in a basal MS medium prepared using the methodology developed by [16]. The MS [72] basal medium was prepared using macronutrients, micronutrients, vitamins, and organic compounds along with sucrose (30 g/L) and agar (8 g/L) and autoclaved at 121 °C and 15 psi for 20 min to achieve sterilization. The cultures were incubated in the culture chamber at 25 ± 1 °C with a 16/8 h photoperiod of light and dark phases using cool white fluorescent tubes (Philips, Rajarhat, India) at a photon flux of 50–70 mmol/m^2^/s for seed germination and seedling establishment (Figure 10).

### 4.2. Explant Preparation and Culture Conditions

Six-week-old in vitro-grown seedlings were used as a source of explants for the excision of stem segments and leaves. Each treatment was applied to 20 explant samples with three replicates. Sections of leaf explants of approximately 0.5 × 0.5 cm^2^ and stem length of size 0.5 cm were prepared and inoculated in callus-induction medium fortified with various concentrations and combinations of PGRs, (2,4-D, NAA, Picloram, Dicamba, TDZ, BAP, IBA, and kinetin), carbon source, (maltose, sucrose, and dextrose), organic adjuvants (yeast extract, casein hydrolysate, and coconut water), and heavy metals, particularly aluminum sulfate, nickel chloride, and ferrous sulfate (Sisco Research Laboratories Pvt. Ltd., Mumbai, India). For explant inoculation, a similar procedure was followed to that applied for in vitro seed germination.

### 4.3. Callus Induction and Plantlet Regeneration

Screw-capped glass bottles (Borosilicate, Delhi, India) of size 300 mL were taken, containing 50 mL of MS medium, and the culture conditions were the same as described above. Plant growth regulators (Himedia Laboratories Pvt. Ltd., Thane, India) were added at various concentrations and combinations. Similar adjustments were made to the organic adjuvants using yeast extract, casein hydrolysate, and coconut water (Himedia Laboratories Pvt. Ltd., Thane, India) and carbon sources, viz., maltose, dextrose, and sucrose (Himedia Laboratories Pvt. Ltd., Thane, India). To assess the response to heavy metals, Fe_2_(SO_4)3_ at 100, 200, and 400 ppm, Al_2_(SO_4_)_3_ at 30, 60, and 90 ppm, and NiCl_2_ (Himedia Laboratories Pvt. Ltd., Thane, India) at 50, 100, 150, 200, 400, and 600 ppm were added to 2,4-D and NAA (Himedia Laboratories Pvt. Ltd., Thane, India) at concentrations of 1.0 and 0.50 mg/L. The nutrient medium without any PGR served as the “control”. Seed sterilization and incubation were performed as per the methodology developed by [16]. The healthy friable embryogenic calli were incubated in MS basal medium supplemented with regeneration medium for 4–6 weeks, respectively, to estimate the callus-induction percentage, number of plantlets per callus, number of roots per responding callus, and shoot length (cm).

### 4.4. Data Analysis

A completely randomized design (CRD) was laid out for the laboratory experiment, each of which comprised three replications and 20 explants per replication. Collected data were statistically subjected to standard statistical methods of analysis of variance (ANOVA) using Agres Statistical Software (c) 1994 Pascal Intl Software Solutions, Version 3.01, and significant differences were compared by LSD at *p* = 0.05 and determined by Duncan’s Multiple Range Test. The analyzed data were used to interpret the results and draw conclusions. The graph was constructed online in RAW Graphs using the website https://app.rawgraphs.io/ accessed on 12 September 2025.

## 5. Conclusions

From this study, a protocol was standardized for indirect regeneration that eventually ensured a foundation for callus induction and plantlet regeneration for further production of true-to-type and disease-free planting material. The frequency of callus induction was maximized by the addition of 2,4-D and Picloram. in response to carbon sources, viz., 30 g/L sucrose and 60 g/L dextrose, and organic adjuvants, viz., coconut water at 10 mL/L and 15 mL/L, enhanced callus induction. The addition of 200 mg/L casein hydrolysate in the callus-induction medium induced the maximum number of shoots per callus. Higher concentrations of Fe_2_(SO_4_)_3_ at 400 ppm enhanced callus induction, respectively. Pearson correlation analysis exhibited a positive and significant relationship regarding the number of plantlets/calluses with shoot length (cm) in *C. jambhiri*. In *C. aurantifolia*, the number of plantlets/calluses showed a positive and significant association with regeneration percentage. Thus, *C. jambhiri* exhibited higher efficiency in producing calluses and plantlet regeneration compared to *C. aurantifolia*. Thus, these protocols will be useful for the commercial propagation system along with micrografting techniques, slow propagule growth, repeated subculture, root culture, anther or microspore culture, and genetic modification for quality parameters, viz., quality retention and sweetness, in future research approaches.

## Figures and Tables

**Table 1:** Effect of organic adjuvants for callus induction and regeneration from mature seeds of *C. jambhiri* and *C. aurantifolia*.

Treatments	Germination (%)	Callus Induction Ability (%)	Regeneration (%)	No. of Plantlets/ Callus	No. of Roots/Responded Callus	Shoot Length (cm)
S1T1	76.36 cd	42.85 c	33.33 e	5.50 a	2.11 cd	2.96 de
S1T2	74.54 d	29.27 e	66.66 c	3.61 b	2.43 cd	2.38 ef
S1T3	80.00 c	36.36 d	50.00 d	2.17 cd	1.95 de	2.82 de
S1T4	63.15 h	27.77 ef	90.00 a	3.70 b	3.23 b	8.00 a
S1T5	9.26 n	60.00 a	9.37 j	2.66 c	2.33 cd	4.38 b
S1T6	93.00 b	12.90 g	75.00 b	5.25 a	2.81 bc	4.07 bc
S1T7	24.00 l	16.66 g	8.95 j	1.33 ef	1.00 g	3.76 bc
S1T8	52.00 i	33.33 d	17.00 fg	1.75 de	1.75 ef	4.18 b
S1T9	16.00 m	25.00 f	15.31 gh	1.00 f	2.00 de	3.42 cd
S2T1	45.33 j	3.13 h	13.76 hi	2.05 cd	2.63 bc	1.96 fg
S2T2	68.00 fg	2.87 h	10.77 ij	1.92 cd	1.73 fg	2.70 de
S2T3	73.33 de	3.11 h	16.08 gh	1.93 cd	2.05 de	2.29 ef
S2T4	53.33 i	28.75 e	33.75 e	2.17 cd	2.47 cd	2.58 ef
S2T5	98.00 a	3.40 h	74.15 b	5.21 a	8.76 a	3.76 bc
S2T6	33.33 k	2.40 h	20.00 f	5.00 a	2.00 de	2.52 ef
S2T7	64.00 gh	43.75 c	11.23 ij	1.81 de	2.42 cd	1.75 g
S2T8	69.33 ef	44.23 c	9.73 j	2.16 cd	2.23 cd	2.34 ef
S2T9	66.66 fg	52.00 b	10.48 j	2.21 cd	2.43 cd	2.45 ef
Mean	58.87	25.99	31.42	2.86	2.57	3.24
Range	9.26–98.00	2.40–60.00	8.95–90.00	1.00–5.21	1.00–8.76	1.75–8.00

Values bearing same letter in the column are not significantly different at *p* = 0.01 of LSD. S1: *C. jambhiri*; S2: *C. aurantifolia*; T1: Yeast extract 200 mg/L; T2: Yeast extract 400 mg/L; T3: Yeast extract 600 mg/L; T4: Casein hydrolysate 50 mg/L; T5: Casein hydrolysate 100 mg/L; T6: Casein hydrolysate 200 mg/L; T7: Coconut water 5 mL/L; T8: Coconut water 10 mL/L; T9: Coconut water 15 mL/L.

**Table 2:** Effect of carbon sources for callus induction and regeneration from matured seeds of *C. jambhiri*.

Treatments /Explants	Germination (%)	Callus-Induction Ability (%)	Regeneration (%)	No. of Plantlets/Callus	No. of Roots/Responding Callus	Shoot Length (cm)
S1T1	48.80 d	16.39 ef	31.15 c	2.30 fg	2.48 bc	4.34
S1T2	47.20 d	13.55 f	59.32 b	3.75 de	1.86 d	2.88
S1T3	20.20 f	25.00 c	16.32 e	1.00 g	1.50 f	3.40
S1T4	3.51 g	50.00 a	14.53 ef	1.00 g	1.00 f	4.50
S1T5	65.00 c	17.95 de	66.66 a	7.85 b	3.13 a	4.38
S1T6	98.00 a	6.12 g	63.26 ab	9.33 a	3.28 a	4.35
S2T1	77.33 b	18.96 de	9.34 g	2.54 ef	2.91 ab	2.83
S2T2	40.00 e	16.66 e	11.27 fg	6.46 c	2.50 bc	1.91
S2T3	53.33 d	20.00 d	8.33 g	4.00 d	2.46 bc	2.99
S2T4	46.66 de	8.57 g	7.67 g	2.33 fg	1.90 d	2.53
S2T5	69.33 c	40.38 b	24.28 d	1.75 fg	2.00 cd	2.57
S2T6	65.33 c	8.16 g	9.61 g	1.71 fg	2.25 cd	3.17
Mean	52.89	20.15	26.81	3.67	2.27	3.32
Range	3.51–98.00	6.12–50.00	9.34–66.66	1.00–9.33	1.00–3.28	NS

Values bearing the same letter in the column are not significantly different at *p* = 0.01 of LSD. S1: *C. jambhiri*; S2: *C. aurantifolia*; T1: Maltose 3%; T2: Maltose 6%; T3: Dextrose 3%; T4: Dextrose 6%; T5: Sucrose 3%; T6: Sucrose 6%.

**Table 3:** Effect of heavy metals on callus induction and regeneration from mature seeds of *C. jambhiri* and *C. aurantifolia*.

Treatments/Explants	Germination %	Callus-Induction Ability %	Regeneration %	No. of Plantlets/Callus	No. of Roots/Responder Callus	Shoot Length (cm)
S1T1	98.00 a	6.12 jk	34.69 cd	13.33 a	1.74 cd	5.00 a
S1T2	45.19 jk	8.00 ij	32.00 de	3.00 cd	1.70 cd	4.78 a
S1T3	15.55 o	50.00 c	13.86 jk	5.00 b	2.73 cd	3.69 bc
S1T4	51.75 i	9.37 ij	9.38 lmn	3.40 c	6.47 ab	2.55 de
S1T5	56.64 gh	8.48 ij	9.08 mn	1.96 fg	3.11 c	2.67 de
S1T6	38.91 lm	8.04 ij	8.61 mn	2.04 fg	3.37 c	2.54 de
S1T7	63.18 ef	7.23 ij	8.01 mn	2.10 fg	2.94 c	2.75 de
S1T8	61.33 fg	5.18 kl	7.56 mn	2.36 de	2.76 cd	2.33 f
S1T9	67.82 de	5.32 kl	6.17 n	2.37 de	2.81 cd	2.51 de
S1T10	37.50 m	20.00 fg	40.00 ab	1.80 fg	2.36 cd	4.97 a
S1T11	66.66 de	5.00 kl	10.00 kl	2.16 ef	2.54 cd	5.00 a
S1T12	77.55 c	2.63 l	36.84 bc	2.19 ef	2.44 cd	2.60 de
S1T13	83.78 b	83.00 b	13.12 jk	3.20 cd	7.50 a	2.63 de
S2T1	70.66 d	6.12 jk	43.77 a	3.00 de	1.00 d	2.50 ef
S2T2	68.00 de	8.00 ij	32.00 e	2.37 cd	6.64 ab	3.86 b
S2T3	77.00 c	50.00 c	19.83 h	2.26 ef	6.72 ab	5.35 a
S2T4	40.00 lm	30.28 d	29.55 e	3.40 c	5.38 b	3.52 bc
S2T5	42.66 kl	26.92 de	25.37 fg	1.96 fg	2.10 cd	2.55 de
S2T6	28.00 n	23.58 ef	24.46 g	2.04 fg	2.43 cd	2.20 f
S2T7	49.33 ij	19.79 g	21.75 gh	2.10 fg	2.50 cd	2.41 ef
S2T8	42.66 kl	15.16 h	18.67 hi	2.36 de	2.18 cd	2.55 de
S2T9	45.33 jk	9.67 ij	15.42 ij	2.37 def	2.12 cd	2.67 de
S2T10	37.50 m	23.85 e	34.92 cd	1.27 g	1.87 cd	3.08 cd
S2T11	56.82 gh	10.70 i	11.37 kl	1.53 fg	2.13 cd	3.25 bc
S2T12	59.03 fg	8.31 ij	29.17 ef	1.65 fg	2.09 cd	2.26 f
S2T13	53.78 hi	54.03 a	34.92 cd	1.27 g	5.60 b	2.62 def
Mean	55.18	19.41	21.94	2.79	3.26	3.19
Range	15.55–98.00	2.63–83.00	6.17–43.77	1.27–13.33	1.00–7.50	2.20–5.35

Values bearing same letter in the column are not significantly different at *p* = 0.01 of LSD. S1: *C. jambhri*; S2: *C. aurantifolia*. T1: Fe_2_SO_4_ 100 ppm; T2: Fe_2_SO_4_ 200 ppm; T3: Fe_2_SO_4_ 400 ppm; T4: NiCl_2_ 50 ppm; T5: NiCl_2_ 100 ppm; T6: NiCl_2_ 150 ppm; T7: NiCl_2_ 200 ppm; T8: NiCl_2_ 400 ppm; T9: NiCl_2_ 600 ppm; T10: Al_2_(SO_4_)_3_ 30 ppm; T11: Al_2_(SO_4_)_3_ 60 ppm; T12: Al_2_(SO_4_)_3_ 90 ppm; T13: Control (1.0 mg/L 2,4-D + NAA @ 0.50 mg/L).

## Data Availability

Data will be available based on the request of the readers.

## Supplementary Materials

The following supporting information can be downloaded at https://www.mdpi.com/article/10.3390/plants15111677/s1, Table S1: Analysis of variance of different tissue culture parameters of *C. Jambhiri* and *C. aurantifolia* under varying combinations of organic adjuvants; Table S2: Effect of explants on regeneration of *C. jambhiri* as influenced by plant growth regulators in the callus induction medium; Table S3: Effect of tissue culture responses of *C.jambhiri* across different treatments; Table S4: Effect of explants on regeneration of *C. aurantifolia* as influenced by plant growth regulators in the callus induction medium; Table S5: Effect of treatments on callus induction from different explants of *C. aurantifolia*; Table S6: Effect of plant growth regulators on plantlets regeneration from matured seed derived calli of *C. jambhiri*; Table S7: Analysis of variance of different tissue culture parameters of *C. jambhiri* and *C. aurantifolia* under varying combinations of organic adjuvants; Table S8: Effect of treatments among the species on tissue cultural response of matured seeds of *C. jambhiri* and *C. aurantifolia* under different medium composition; Table S9: Effect of species on germination of seeds among the treatments under different medium composition; Table S10: Analysis of variance of different tissue culture parameters of *C. jambhiri* and *C. aurantifolia* under varying combinations of carbon sources; Table S11: Effect of carbon sources for callus induction and regeneration from matured seeds of *C. jambhiri*; Table S12: Effect of treatments among the species on germination of matured seeds of *C. jambhiri* and *C. aurantifolia* under different medium compositions; Table S13: Effect of species among the treatments on germination of matured seeds of *C. jambhiri* and *C. aurantifolia* under different medium compositions; Table S14: Analysis of variance of different tissue culture parameters of *C. jambhiri* and *C. aurantifolia* under varying combinations of carbon sources; Table S15: Effect of heavy metals and its concentrations among the species on tissue cultural response of matured seeds of *C. jambhiri* and *C. aurantifolia*; Table S16: Effect of species among the metals and its concentrations on tissue cultural responses of matured seeds of *C. jambhiri* and *C. aurantifolia*.

## Abbreviations

The following abbreviations are used in this manuscript:

FeSO_4_	Ferrous sulfate
Al_2_SO_4_	Aluminum sulfate
NiCl_2_	Nickel chloride
2,4-D	2,4-Dichlorophenoxyacetic acid
NAA	Naphthalene acetic acid
BAP	6-Benzylaminopurine
IBA	Indole-3-butyric acid
TDZ	Thidiazuron
