# Supplementary material for: In Vitro Regeneration Strategies in Woody Citrus of Northeast India (Citrus jambhiri and Citrus aurantifolia)
Source: Plants (Basel). 2026 May 29;15(11):1677. doi: 10.3390/plants15111677 (PMC13259310; doi:10.3390/plants15111677)
Supplement: Supplementary file 1 [file plants-15-01677-s001.zip › plants-3853274-supplementary.pdf]

Table S1. Analysis of variance of different tissue culture parameters of *C. Jambhiri* and *C. aurantifolia* under varying combinations of organic adjuvants

**Source**	**d.f.**	**Mean sum of square**				
		Callus induction	Regeneration	No. of shoots/callus	No. of roots/shoot	Shoot length
Total	59	643.245	423.735	1.047	4.497	0.469
Treatment	29	1300.058^{NS}	857.630^{NS}	2.037^{NS}	9.063^{NS}	0.900^{NS}
Error	30	8.325	4.303	0.090	0.084	0.053
Explant(E)	2	6974.571**	960.340**	9.348**	11.783**	4.139**
Treatments(T)	9	809.517**	1285.229**	1.823**	16.001**	0.467**
E×T	18	914.826**	632.419**	1.331**	5.291**	0.756**
Error	30	8.325	4.303	0.090	0.084	0.053

**denote significance P=0.01; NS: Non-significant

Table S2. Effect of explants on regeneration of *C. jambhiri* as influenced by plant growth regulators in the callus induction medium

Explants	Tissue cultural responses				
	Callus induction (%)	Regeneration (%)	No. of explant	shoot/No. of root/ shoot	Shoot length (cm)
S1	53.17c	36.12a	2.84a	3.36a	3.33a
S2	82.15b	36.31a	1.98b	2.00b	2.59b
S3	88.07a	24.21b	1.49c	2.07b	2.50b
Mean	74.46	32.21	2.10	2.48	2.81

Values bearing same letter in the column are not significantly different at p = 0.01 of LSD

S1: Matured seeds; **S2:** Plumules (stem); **S3:** Leaf

Table S3. Effect of tissue culture responses of *C.jambhiri* across different treatments

Treatments	Tissue cultural responses				
	Callus induction (%)	Regeneration (%)	No. of shoot/ explant	No. of root/ shoot	Shoot length (cm)
T1	64.33e	25.88de	1.90cd	2.35c	3.15ab
T2	59.13f	38.26c	2.03bc	2.36c	2.97abc
T3	94.33a	23.67ef	2.29b	6.86a	2.74cde
T4	84.17b	59.72a	2.37b	3.27b	2.60e
T5	58.99f	16.93g	1.85cd	1.60de	2.87cde
T6	80.87b	24.92ef	1.78cd	1.80d	2.30f
T7	83.69b	25.64de	1.69cd	1.63de	2.67de
T8	75.23c	23.16f	1.67d	1.38e	2.63e
T9	69.86d	56.45b	3.52a	1.82d	3.24a
T10	74.04c	27.51d	1.90cd	1.72de	2.90bcd
Mean	74.46	32.21	2.10	2.48	2.81
Range	58.99-94.33	16.93-59.72	1.90-3.52	1.38-6.86	2.63-3.24

Values bearing same letter in the column are not significantly different at p = 0.01 of LSD
T1: 2,4-D 1.0 mg/L; **T2:** 2,4-D 2.0 mg/L; **T3:** 2,4-D 1.0 mg/L + NAA 0.50 mg/L; **T4:** 2,4-D 2.0 mg/L + NAA
0.50 mg/L; **T5:** Picloram 0.50 mg/L; **T6:** Picloram 1.0 mg/L; **T7:** Dicamba 0.50 mg/L; **T8:** Dicamba 1.0mg/L; **T9:** TDZ 0.25 mg/L; **T10:** TDZ 0.50 mg/L

Table S4. Effect of explants on regeneration of *C. aurantifolia* as influenced by plant growth regulators in the callus induction medium

Explant	Tissue cultural responses				
	Callus induction (%)	Regeneration (%)	No. of shoot/ explant	No. of root/ shoot	Shoot length (cm)
S1	53.82c	35.53a	1.53b	3.92a	3.67a
S2	87.34b	32.68b	1.73a	2.28c	2.16b
S3	90.75a	20.47c	1.32c	2.66b	2.03b
**Mean**	**77.30**	**29.56**	**1.53**	**2.96**	**2.62**

Values bearing same letter in the column are not significantly different at $p = 0.01$ of LSD

S1: Matured seeds; **S2:** Plumules (stem); **S3:** Leaf

Table S5. Effect of treatments on callus induction from different explants of *C. aurantifolia*

Treatments	Tissue cultural responses				
	Callus induction (%)	Regeneration (%)	No. of shoot/ explant	No. of root/ shoot	Shoot length (cm)
T1	82.15ab	34.03d	1.68bc	6.24a	4.32a
T2	75.82d	42.77c	1.98a	2.59d	3.25b
T3	80.51bc	30.94e	1.81ab	5.39b	2.14c
T4	79.42c	47.27b	1.85ab	2.70d	2.20c
T5	83.42a	10.70h	1.27ef	1.45fg	2.35b
T6	80.95abc	10.27h	1.23ef	1.33g	3.13c
T7	71.92e	27.52f	1.42de	3.29c	2.26c
T8	71.28e	15.17g	1.10f	2.42d	2.28c
T9	65.60f	60.38a	1.49cd	2.25de	2.21c
T10	81.91abc	16.54g	1.43de	1.89ef	2.04c
Mean	77.30	29.56	1.53	2.96	2.62
Range	65.60-83.42	15.17-60.38	1.10-1.98	1.33-6.24	2.04-4.32

Values bearing same letter in the column are not significantly different at $p = 0.01$ of LSD
T1: 2,4-D 1.0 mg/L; **T2:** 2,4-D 2.0 mg/L; **T3:** 2,4-D 1.0 mg/L + NAA 0.50 mg/L; **T4:** 2,4-D 2.0 mg/L + NAA 0.50 mg/L; **T5:** Picloram 0.50 mg/L; **T6:** Picloram 1.0 mg/L; **T7:** Dicamba 0.50 mg/L; **T8:** Dicamba 1.0mg/L; **T9:** TDZ 0.25 mg/L; **T10:** TDZ 0.50 mg/L

Table S6. Effect of plant growth regulators on plantlets regeneration from matured seed derived calli of *C. jambhiri*

Regeneration medium	Regeneration	No. of shoots per callus	No. of roots per shoot
MS+1mg/L BAP+1mg/L IBA	63.73	2.66	2.40
MS+1mg/LBAP+1mg/L Kinetin+0.50mg/L NAA	88.97	3.25	2.50

Table S7. Analysis of variance of different tissue culture parameters of *C. jambhiri* and *C. aurantifolia* under varying combinations of organic adjuvants

Source	d.f.	Mean sum of square					
		Germination	Callus induction	Regeneration	No. of shoots/callus	No. of roots/shoots	Shoot length
Total	53	606.298	334.042	717.258	2.220	2.646	2.093
Treatment	17	1875.800^{NS}	1033.290^{NS}	2229.179^{NS}	6.440^{NS}	7.838^{NS}	6.094^{NS}
Error	36	6.811	3.842	3.296	0.228	0.195	0.203
Species (S)	1	1148.166**	1683.375**	4574.424**	1.050^{NS}	8.425**	30.917**
Treatments (T)	8	789.460**	643.870**	1901.230**	8.888**	8.072**	5.093**
S × T	8	3053.094**	1341.449**	2263.972**	4.665**	7.530**	3.994**
Error	36	6.811	3.842	3.296	0.228	0.195	0.203

** denote significance P = 0.01; NS: Non-significant

Table S8. Effect of treatments among the species on tissue cultural response of matured seeds of *C. jambhiri* and *C. aurantifolia* under different medium composition

Treatments	Tissue cultural responses					
	Germination (%)	Callus induction(%)	Regeneration (%)	No. of shoots/explant	No. of roots/shoot	Shoot length(cm)
T1	60.85cd	22.99d	23.55f	3.78b	2.37bc	2.46e
T2	71.27b	16.07f	38.72d	2.77c	2.08cd	2.54e
T3	76.67a	19.74e	33.04e	2.05d	2.00cd	2.56e
T4	58.24d	28.26c	61.88a	2.94c	2.85b	5.29a
T5	53.63e	31.70b	41.76c	3.94b	5.55a	4.07b
T6	63.17c	7.65g	47.50b	5.13a	2.41bc	3.30c
T7	44.00f	30.21bc	10.09h	1.57d	1.71d	2.76de
T8	60.67cd	38.78a	13.37g	1.96d	1.99cd	3.26cd
T9	41.33f	38.50a	12.90g	1.61d	2.22cd	2.94cde
Mean	58.87	25.99	31.42	2.86	2.57	3.24
Range	41.33-76.67	7.65-38.78	10.09-61.88	1.57-5.13	1.71-5.55	2.54-5.29

Values bearing same letter in the column are not significantly different at $p = 0.01$ of LSD

T1: Yeast extract 200 mg/L; **T2:** Yeast extract 400 mg/L; **T3:** Yeast extract 600 mg/L; **T4:** Casein hydrolysate50 mg/L; **T5:** Casein hydrolysate 100 mg/L; **T6:** Casein hydrolysate 200 mg/L; **T7:** Coconut water 5 ml/L; **T8:** Coconut water 10 ml/L; **T9:** Coconut water 15 ml/L

Table S9. Effect of species on germination of seeds among the treatments under differentmedium composition

Species											
	Tissue cultural responses										
	Germination (%)		Callus induction (%)		Regeneration (%)		No. of shoots/ explant		No. of roots/ shoot		Shoot length (cm)
S1	54.26b		31.57a		40.62a		3.00NS		2.18b		4.00a
S2	63.48a		20.40b		22.22b		2.72NS		2.97a		2.48b
Mean	58.87	-	25.99	-	31.42	-	2.86	-	2.57	-	3.24 -

Values bearing same letter in the column are not significantly different at $p = 0.01$ of LSD

S1: *C. jambhiri*; **S2:** *C. aurantifolia*

Table S10. Analysis of variance of different tissue culture parameters of *C. jambhiri* and *C. aurantifolia* under varying combinations of carbon sources

Source	d.f.	Mean sum of square					
		Germination	Callus induction	Regeneration	No. of shoots/callus	No. of roots/shoot	Shoot length
Total	35	608.967	162.983	501.981	7.646	0.493	0.809
Treatment	11	1899.596 ^{NS}	511.268^{NS}	1584.540 ^{NS}	22.947 ^{NS}	1.332 ^{NS}	2.280 ^{NS}
Error	24	17.428	3.353	5.807	0.633	0.109	0.134
Species (S)	1	1199.583**	66.259**	8166.736**	10.368**	0.148^{NS}	15.405**
Treatments (T)	5	2705.693**	445.294**	1215.059**	16.452**	1.532**	1.436**
S ×T	5	1233.502**	666.244**	637.582**	31.959**	1.369**	0.500 ^{NS}
Error	24	17.428	3.353**	5.807	0.633	0.109	0.134

** denote significance P = 0.01; NS: Non-significant

Table S11. Effect of carbon sources for callus induction and regeneration from matured seeds of *C. jambhiri*.

Treatments / Explants	Germination (%)	Callus induction ability (%)	Regeneration (%)	No. of plantlets/ callus	No. of roots/ responded callus	Shoot length(cm)
S1T1	48.80d	16.39ef	31.15c	2.30fg	2.48bc	4.34
S1T2	47.20d	13.55f	59.32b	3.75de	1.86d	2.88
S1T3	20.20f	25.00c	16.32e	1.00g	1.50f	3.40
S1T4	3.51g	50.00a	14.53ef	1.00g	1.00f	4.50
S1T5	65.00c	17.95de	66.66a	7.85b	3.13a	4.38
S1T6	98.00a	6.12g	63.26ab	9.33a	3.28a	4.35
S2T1	77.33b	18.96de	9.34g	2.54ef	2.91ab	2.83
S2T2	40.00e	16.66e	11.27fg	6.46c	2.50bc	1.91
S2T3	53.33d	20.00d	8.33g	4.00d	2.46bc	2.99
S2T4	46.66de	8.57g	7.67g	2.33fg	1.90d	2.53
S2T5	69.33c	40.38b	24.28d	1.75fg	2.00cd	2.57

S2T6	65.33c	8.16g	9.61g	1.71fg	2.25cd	3.17
Mean	52.89	20.15	26.81	3.67	2.27	3.32
Range	3.51-98.00	6.12-50.00	9.34-66.66	1.00-9.33	1.00-3.28	NS

Values bearing same letter in the column are not significantly different at p = 0.01 of LSD

S1: *C. jambhiri*; **S2:** *C. aurantifolia*

T1: Maltose 3%; **T2:** Maltose 6%; **T3:** Dextrose 3%; **T4:** Dextrose 6%; **T5:** Sucrose 3%; **T6:** Sucrose 6%

Table S12. Effect of treatments among the species on germination of matured seeds of *C. jambhiri* and *C. aurantifolia* under different medium compositions

Treatments	Tissue cultural responses					
	Germination (%)	Callus induction(%)	Regeneration (%)	No. of shoot/ explant	No. of roots/shoot	Shoot length (cm)
T1	63.07b	17.68c	20.25c	2.42b	2.70a	3.59ab
T2	43.60c	15.11d	35.30b	5.11a	2.18bc	2.40c
T3	36.77d	22.50b	12.33d	2.50b	1.98c	3.20b
T4	25.09e	29.29a	11.10d	1.67b	1.45d	3.52ab
T5	67.17b	29.17a	45.47a	4.80a	2.57ab	3.48ab
T6	81.67a	7.14e	36.44b	5.52a	2.77a	3.76a
Mean	52.89	20.15	26.81	3.67	2.27	3.32
Range	25.09-81.67	7.14-29.29	11.10-45.47	1.67-5.52	1.45-2.77	2.40-3.76

Values bearing same letter in the column are not significantly different at p = 0.01 of LSD

T1: Maltose 3%; **T2:** Maltose 6%; **T3:** Dextrose 3%; **T4:** Dextrose 6%; **T5:** Sucrose 3%; **T6:** Sucrose 6%

Table S13: Effect of species among the treatments on germination of matured seeds of *C. jambhiri* and *C. aurantifolia* under different medium compositions

Species	Tissue cultural responses					
	Germination (%)	Callus induction (%)	Regeneration (%)	No. of shoot/ explant	No. of roots/ shoot	Shoot length(cm)
S1	47.12b	21.50a	41.87a	4.21a	2.21	3.98a
S2	58.66a	18.79b	11.75b	3.13b	2.34	2.67b
Mean	52.89-	20.15-	26.81-	3.67-	NS	3.32-

Values bearing same letter in the column are not significantly different at p = 0.01 of LSD

S1: *C. jambhiri*; **S2:** *C. aurantifolia*

Table S14. Analysis of variance of different tissue culture parameters of *C. jambhiri* and *C. aurantifolia* under varying combinations of carbon sources

Source	d.f.	Mean sum of square					
		Germination	Callus induction	Regeneration	No. of shoots/callus	No. of roots/shoot	Shoot length
Total	35	333.581	379.715	137.542	5.219	3.694	4.713
Treatment	11	674.633 ^{NS}	771.171 ^{NS}	276.828 ^{NS}	10.468 ^{NS}	6.721 ^{NS}	8.576 ^{NS}
Error	24	5.646	3.316	3.612	0.173	0.783	1.000
Species (S)	1	666.596**	356.110**	962.856**	23.102**	0.006- ^{NS}	0.008**
Treatments (T)	5	748.619**	1422.118**	409.972**	11.867**	9.910**	12.645**
S × T	5	601.318**	154.812**	86.515**	8.017**	4.091**	5.221**

Error	24	5.646	3.316	3.612	0.173	0.783	1.000

** denote significance P = 0.01; NS: Non-significant

Table S 15. Effect of heavy metals and its concentrations among the species on tissue cultural response of matured seeds of *C. jambhiri* and *C. aurantifolia*

Treatments	Tissue cultural responses					
	Germination (%)	Callus induction	Regeneration (%)	No. of shoot/ explant	No. of root/ shoot	Shoot length (cm)
T1	84.33a	6.12h	39.23a	8.17a	1.12f	3.75b
T2	56.60d	8.00gh	32.00b	2.69c	4.17cd	4.32a
T3	46.28fg	50.00b	16.85de	3.63b	4.73bc	4.52a
T4	45.88g	19.83cd	19.47de	3.40b	5.93ab	3.04c
T5	49.65ef	17.70de	17.23d	1.96de	2.61ef	2.61cd
T6	33.46i	15.81ef	16.54e	2.04de	2.90de	2.37d
T7	56.26d	13.51f	14.88ef	2.10cde	2.72e	2.58cd
T8	52.00e	10.17g	13.12fg	2.36cd	2.47ef	2.44d
T9	56.58d	7.50h	10.80g	2.37cd	2.47ef	2.59cd
T10	37.50h	21.93c	37.46a	1.54e	2.12ef	4.03ab
T11	61.74c	7.85gh	10.69g	1.85de	2.34ef	4.13ab
T12	68.29b	5.47h	33.01b	1.92de	2.27ef	2.43d
T13	68.78b	68.52a	24.02c	2.24cd	6.55a	2.63cd
Mean	55.18	19.41	21.94	2.79	3.26	3.19
Range	33.46-84.33	5.47-68.52	10.80-39.23	1.96-8.17	1.12-6.55	2.37-4.52

*Control: MS + 1.0 mg/L of 2,4-D + NAA @ 0.50 mg/L

Values bearing same letter in the column are not significantly different at p = 0.01 of LSD

T1: Fe_2SO_4 100 ppm; **T2:** Fe_2SO_4 200 ppm; **T3:** Fe_2SO_4 400 ppm; **T4:** $NiCl_2$ 50 ppm; **T5:** $NiCl_2$ 100 ppm; **T6:** $NiCl_2$ 150 ppm; **T7:** $NiCl_2$ 200 ppm; **T8:** $NiCl_2$ 400 ppm; **T9:** $NiCl_2$ 600 ppm; **T10:** Al_2SO_4 30 ppm; **T11:**Al_2SO_4 60 ppm; **T12:** Al_2SO_4 90 ppm; **T13:** Control (1.0 mg/L of 2,4-D + NAA @ 0.50 mg/L)

Table S 16. Effect of species among the metals and its concentrations on tissue cultural responsesof matured seeds of *C. jambhiri* and *C. aurantifolia*

Species	Germination (%)	Callus induction (%)	Regeneration (%)	No. of shoot/ explant	No. of root/ shoot	Shoot length (cm)
S1	58.76a	16.80b	17.64b	3.45a	3.27	5.00a
S2	51.60b	22.03a	26.25a	2.12b	3.25	2.50b
Mean	55.18	19.41	21.94	2.79	NS	3.75-

Values bearing same letter in the column are not significantly different at p = 0.01 of LSD

S1: *C. jambhri*; **S2:** *C. aurantifolia*
